# Transillumination Test as a Bedside Screening and Assessment Tool in Neonates and Infants with Hydrocephalus

**DOI:** 10.21315/mjms-11-2024-900

**Published:** 2025-02-28

**Authors:** Karrthik Murugasan, Eddie Lim Wei Ming, Lee Calwin, Balamurugan Rajendran

**Affiliations:** 1 Department of Neurosciences, School of Medical Sciences, Universiti Sains Malaysia, Health Campus, Kelantan, Malaysia; 2 Hospital Pakar Universiti Sains Malaysia, Universiti Sains Malaysia, Health Campus, Kelantan, Malaysia; 3 Department of Neurosurgery, Hospital Umum Sarawak, Sarawak, Malaysia; 4 Department of Neurosurgery, Hospital Queen Elizabeth, Sabah, Malaysia

Dear Editor,

We recently came across an intriguing article by Zakaria et al. (1), in which the authors provided detailed descriptions and illustrated videos of three clinical neonatal and neurological assessments.

Congenital hydrocephalus is a condition that is characterised by an excessive accumulation of cerebrospinal fluid (CSF) in the ventricles of the brain (2). It can lead to increased intracranial pressure and subsequent neurological impairment. This condition is present at birth and can be caused by a variety of factors, including genetic predisposition, intrauterine infections, intraventricular haemorrhage in the fetus and developmental disorders such as neural tube defects (3).

Hydrocephalus continues to be one of the most prevalent childhood neurological disorders worldwide, with a reported prevalence of between 1 and 32 per 10,000 births (3). The pathophysiology of congenital hydrocephalus often relates to an imbalance between the production and absorption of CSF or to an obstruction within the ventricular system that impedes normal CSF flow (4). The resulting expansion of the ventricles can lead to the compression of the surrounding brain tissue. It can also lead to a spectrum of clinical manifestations ranging from head enlargement to developmental delay, seizures and other neurological deficits.

In Malaysia, congenital hydrocephalus still poses significant clinical challenges and affects long-term neurodevelopmental outcomes. Using NeroLux^©^. We want to demonstrate an innovative examination of a macrocephalic child. Regarding the techniques and tools that are needed for bedside screening and assessment, we also wish to further elaborate on the transillumination test. It is described by Zakaria et al. (1) in their neurological examination of the head of an infant with suspected hydrocephalus.

As early as 1831, the illumination of the cranial cavity was initially described by Richard Bright, who used sunlight and candlelight in his research. He explained that the cranium appeared semi-transparent (5). In this process, the fontanelles in infants allow for high-quality illumination into the cranial cavity. Transillumination refers to light that passes through the cranial cavity and illuminates the opposite side (5). The extent of the glow on the scalp around the rim of a flashlight or torch is then recorded for the interpretation of the results. Transillumination tests can be a cheap and easy screening method for the detection of early congenital hydrocephalus, and they allow for earlier investigation and intervention to improve long-term neurological outcomes. Our proposed examination technique has been depicted by Dodge et al. (5). The authors introduced a simple yet safe transillumination technique, and they identified the factors that could affect the illumination and its results.

## Tool NeroLux^©^

Nero (νɛρó) is the Greek word for water, and lux is the Latin word for light. The light source should be bright, with minimal light escape and heat dissipation to prevent harm to the patient’s head or scalp. The tip of the light source that touches the infant’s head should be soft to ensure manoeuvrability and to prevent trauma to the scalp. We have thus created a self-assembled tool (NeroLux^©^, copyright number: CRLY2025P00274). To ensure maximal brightness along with minimal heat dissipation, it is composed of a light-emitting diode (LED) torch of 150 lux rather than a tungsten filament (6). It also includes a rubber cap, which is secured with black tape on the illumination end to minimise the escape of light and to prevent trauma to the infant’s scalp. The NeroLux^©^ is also small in size and lightweight, allowing easy portability for clinicians to use during a bedside examination. A physiological illuminance would be not more than 3 cm from the rim of the rubber cap.

Diaphanoscopy is the act of using a bright light to shine through tissue. Tansillumination of the skull occurs when there is increased space between the skull and brain, with light being refracted and transmitted through an increased volume of subdural or subarachnoid fluid. It has been shown by Dodge et al. (5) that physiological illuminance does not exceed 2.5 cm beyond the rim of the flashlight and by Barozzino et al.(7) that more than 2 cm around the rim or asymmetry suggests underlying pathology. It has been proven to be an effective examination method in all infants under 12 months of age with a skull thickness of 2.5 mm or less. There have been four key factors that have been proven to affect or modify the transillumination, which should be taken into consideration when conducting the examination. Namely, the hair, the scalp, the bone, and the nature of fluid within the skull (5). Factors that may cause increased transillumination are sparse and blonde or light-coloured hair, fair and fatty scalp, thin skull bone, and clear fluid within the skull. Factors that may decrease transillumination are thick, curly, black hair, dark scalp or presence of scalp hematoma, thick skull bone, and bloody or turbid fluid within the skull.[Fig f1-18mjms3201_le][Fig f2-18mjms3201_le][Fig f3-18mjms3201_le]

## Method of Examination

This clinical method currently practiced by all Malaysian neurosurgical graduates using NeroLux^©^, adopted officially since the end of 2024, has a sensitivity of 90.91% (95% CI: 70.84% to 98.88%) and a specificity of 100% (95% CI: 84.56% to 100.00%) compared to the old method of black rolled paper with transillumination which has a sensitivity of 27.27% (95% CI: 10.73% to 50.22%) and a specificity of 45.45% (95% CI: 24.39% to 67.79%). The positive predictive value (PPV) is 33.33% (95% CI: 18.62% to 52.21%), and the negative predictive value (NPV) is 38.46% (95% CI: 27.00% to 51.36%).

Positive Predictive Value (PPV):

- Current: 100% (95% CI: 83.16% to 100.00%)
- Previous: 33.33% (95% CI: 18.62% to 52.21%)

The current NeroLux^©^ illumination test shows perfect positive predictive value—it always correctly identifies people with hydrocephalus. The previous test was wrong two-thirds of the time.

Negative Predictive Value (NPV):

- Current: 91.67% (95% CI: 74.58% to 97.63%)
- Previous: 38.46% (95% CI: 27.00% to 51.36%)

The current test is highly reliable in ruling out hydrocephalus when negative, while the previous test’s negative results were unreliable.

The improved accuracy of using the NeroLux^©^ is reflected in the overall accuracy statistic:

- Current: 95.45% (95% CI: 84.53% to 99.44%)
- Previous: 36.36% (95% CI: 22.41% to 52.23%)

## Conclusion

We have created and demonstrated a simple, cheap, and effective method using NeroLux^©^ to screen infants or children under two years for congenital abnormalities and abnormal cerebral fluid accumulation. The estimated cost of assembling NeroLux^©^ is around RM 20. This can allow doctors in developing countries lacking resources to effectively identify congenital or acquired pathologies and enable further investigation and treatment. Transillumination has been present for decades. In this modern era, this examination method can be cultivated to improve screening methods and possibly reduce the morbidity of paediatric patients in the future with early detection and intervention.

## Figures and Tables

**Figure 1 f1-18mjms3201_le:**
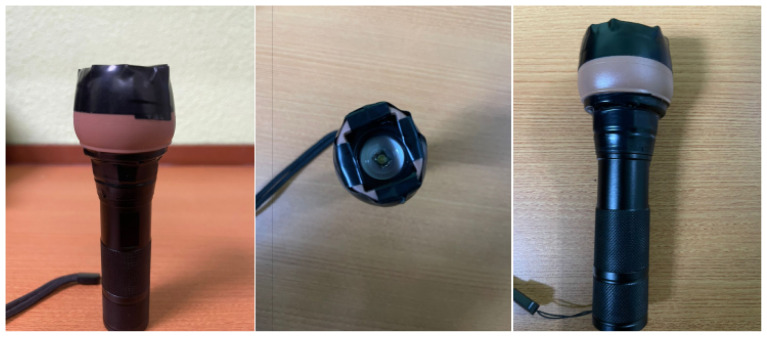
NeroLux^©^

**Figure 2 f2-18mjms3201_le:**
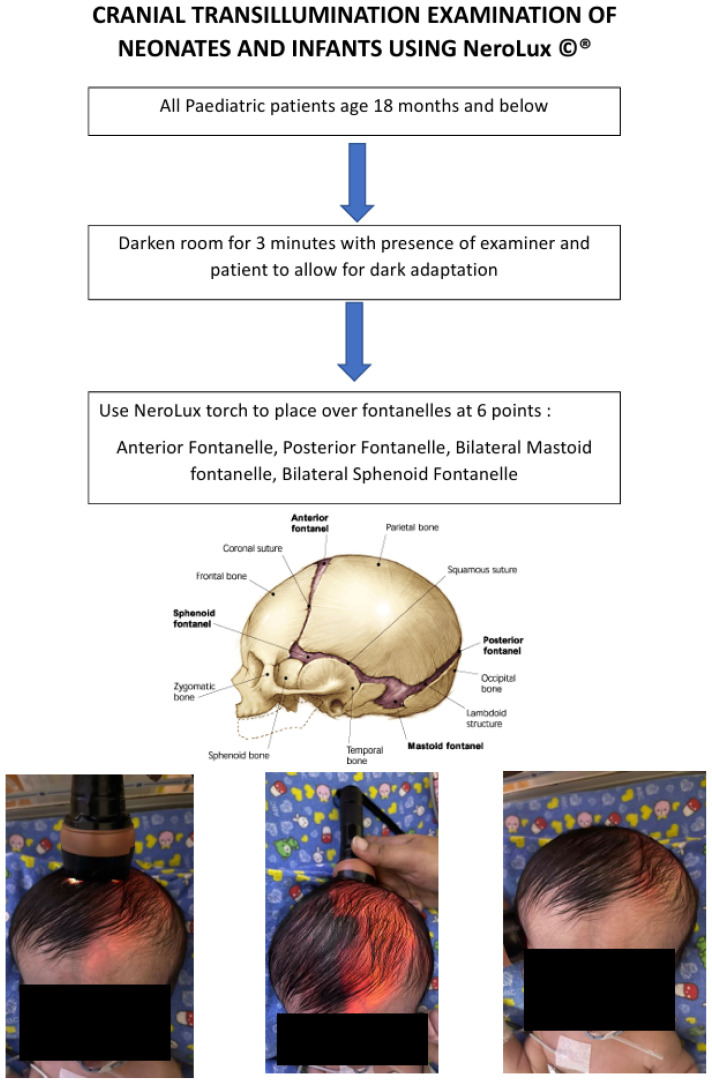
Method of examination

**Figure 3 f3-18mjms3201_le:**
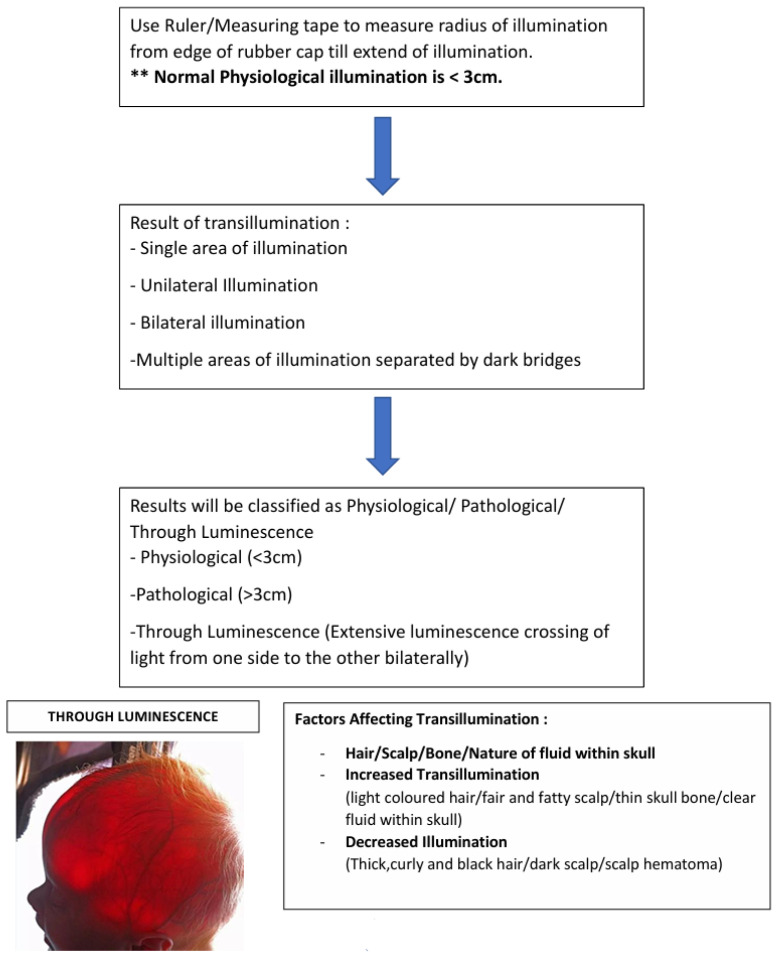
Interpretation of results
